# Immune-related lncRNAs as predictors of survival in breast cancer: a prognostic signature

**DOI:** 10.1186/s12967-020-02522-6

**Published:** 2020-11-23

**Authors:** Wei Ma, Fangkun Zhao, Xinmiao Yu, Shu Guan, Huandan Suo, Zuo Tao, Yue Qiu, Yunfei Wu, Yu Cao, Feng Jin

**Affiliations:** 1 Department of Breast Surgery, The First Affiliated Hospital of China Medical University, Shenyang, Liaoning Province, China; 2 Department of Ophthalmology, The Fourth Affiliated Hospital of China Medical University, Shenyang, Liaoning Province, China; 3 Department of Cardiovascular Ultrasound, The First Affiliated Hospital of China Medical University, Shenyang, Liaoning Province, China

**Keywords:** Breast cancer, Long non-coding RNA, Immune-related predictors, Prognostic signature, TCGA, Overall survival

## Abstract

**Background:**

Breast cancer is a highly heterogeneous disease, this poses challenges for classification and management. Long non-coding RNAs play acrucial role in the breast cancersdevelopment and progression, especially in tumor-related immune processes which have become the most rapidly investigated area. Therefore, we aimed at developing an immune-related lncRNA signature to improve the prognosis prediction of breast cancer.

**Methods:**

We obtained breast cancer patient samples and corresponding clinical data from The Cancer Genome Atlas (TCGA) database. Immune-related lncRNAs were screened by co-expression analysis of immune-related genes which were downloaded from the Immunology Database and Analysis Portal (ImmPort). Clinical patient samples were randomly separated into training and testing sets. In the training set, univariate Cox regression analysis and LASSO regression were utilized to build a prognostic immune-related lncRNA signature. The signature was validated in the training set, testing set, and whole cohorts by the Kaplan–Meier log-rank test, time-dependent ROC curve analysis, principal component analysis, univariate andmultivariate Cox regression analyses.

**Results:**

A total of 937 immune- related lncRNAs were identified, 15 candidate immune-related lncRNAs were significantly associated with overall survival (OS). Eight of these lncRNAs (OTUD6B-AS1, AL122010.1, AC136475.2, AL161646.1, AC245297.3, LINC00578, LINC01871, AP000442.2) were selected for establishment of the risk prediction model. The OS of patients in the low-risk group was higher than that of patients in the high-risk group (*p* = 1.215e − 06 in the training set; *p* = 0.0069 in the validation set; *p* = 1.233e − 07 in whole cohort). The time-dependent ROC curve analysis revealed that the AUCs for OS in the first, eighth, and tenth year were 0.812, 0.81, and 0.857, respectively, in the training set, 0.615, 0.68, 0.655 in the validation set, and 0.725, 0.742, 0.741 in the total cohort. Multivariate Cox regression analysis indicated the model was a reliable and independent indicator for the prognosis of breast cancer in the training set (HR = 1.432; 95% CI 1.204–1.702, *p* < 0.001), validation set (HR = 1.162; 95% CI 1.004–1.345, *p* = 0.044), and whole set (HR = 1.240; 95% CI 1.128–1.362, *p* < 0.001). GSEA analysis revealed a strong connection between the signature and immune-related biological processes and pathways.

**Conclusions:**

We constructed and verified a robust signature of 8 immune-related lncRNAs for the prediction of breast cancer patient survival.

## Background

Among women, breast cancer is the most commonly diagnosed cancer and the leading cause of cancer-related death [1, 2]. Globally, it is reported that nearly 2.1 million new breast cancer cases were diagnosed, and more than 0.6 million women died of breast cancer in 2018 [3].Breast cancer is a highly heterogeneous disease and, thus, its etiology, pathological manifestations, and outcomes vary from person to person [4, 5]. Considering the high mortality and heterogeneity, it is urgent to identify suitable detection approaches for breast cancer prognosis biomarkers. Many studies have shown that some genes and mRNAs play significant roles as prognostic molecular markers in malignancies [6–8]. Recently, research into tumor immunity has become the most rapidly advancing area within cancer. Immunotherapy provides the unprecedented opportunity to effectively treat malignancies owing to the essential involvement of the immune system in tumor development, progression, and therapy [9], especially in some malignancies such as hepatocellular carcinoma [10], early-stage squamous cell cancer of the anal canal [11], prostate cancer [12].

Long non-coding RNAs (lncRNAs) are classified as transcripts that are longer than 200 nucleotides and do not encode proteins. However, lncRNAs can physically interact with DNA, RNA, or protein. Through such interactions, lncRNAs are able to regulate gene expression at various levels such as transcriptional, post-transcriptional, and translational regulation.As a result, lncRNAs have important roles in the occurrence, progression, and prognosis of cancers and various other diseases [13–15]. In addition, recent studies have indicated that lncRNAs have crucial functions in different phases of cancer immunity such as antigen presentation, immune activation, and immune cell infiltration [16, 17]. Thus, immune-related lncRNAs have drawn considerable attention. One study has reported the prognostic merit of an immune-related lncRNA signature in the prediction of overall survival (OS) in breast cancer [5]. However, this immune-related lncRNA signature has not been validated externally by other researches yet.

In the present study, we identified and validated a robust and reliable molecular signature for the prediction of survival in breast cancer patients. Our results validated a risk scoring model based on 8 immune-related lncRNAs. The model can be used as a reliable prognostic predictor, and the 8 lncRNAs could be potential therapeutic targets for breast cancer.

## Methods

### Data source and preprocessing

We downloaded RNA sequencing data sets as well as the corresponding clinical characteristics of breast cancer samples and normal samples from the TCGA website (https://cancergenome.nih.gov/) in February 2020. Male breast cancer samples or samples with a follow-up time of less than 30 days were excluded. The expression levels were normalized using the Trimmed Mean of M-values method from the “edge” R package and underwent a log2(x + 1) transformation. R3.6.2 software was applied to normalize, process, and analyze the data. Perl (https://www.perl.org/) was used to integrate the RNAseq value of each sample into a matrix file. A list of immune-related genes was downloaded from the gene list resources in Immunology Database and Analysis Portal (ImmPort), an open repositories of subject-level human immunology database for translational and clinical research (https://www.immport.org/) [18].

### Identification of immune-related lncRNAs

Immune-related lncRNAs were screened by co-expression analysis of immune-related genes from the list as described above. All samples were randomly split into training and validation sets using the R software, at a 1:1 ratio. Univariate Cox proportional hazard regression analysis was conducted to screen the immune-related lncRNAs significantly associated with overall survival (OS) in the training cohort with *p* < 0.01 as the criterium. The least absolute shrinkage and selectionoperator (LASSO) regression method was used for the identification of immune-related lncRNAs most correlated with overall survival using the “glmnet” package for R.

### Construction of the immune-related risk prognostic system

Multivariate Cox regression analysis was used to identify significant lncRNAs for construction of the prognostic signature**.** We then calculated the risk score based on the expression levels of lncRNAs for each patient through following formula [19]: Riskscore = exp1*β1 + exp2*β2 … + expi*βi (expi was the expression value of each lncRNA, and βi was the regression coefficient of the multivariate Cox analysis for the target lncRNA). According to the median risk score in the training set, breast cancer patients were divided into a high-risk group and a low-risk group.

### Application and validation of the risk scoring system

In order to validate the predictive value of the model, we performed the Kaplan–Meier log-rank test, time-dependent ROC curve analysis, univariate analysis, and multivariate Cox regression analysis for comparison of the survival between the high- and low-risk group in the training, validation, and the total cohort using the R packages “survival” and “survivalROC”. We then analyzed the correlation between the expression of the 8 immune-related lncRNAs and clinicopathological characteristics.

### Gene set enrichment analysis

In order to identify different functional phenotypes between the high-risk and low-risk groups, we performed a Gene set enrichment analysis (GSEA) 4.0.3(https://www.broadinstitute.org/gsea/index.jsp) [20]. The mRNA expression profiles of breast cancer samples from the TCGA dataset, which were divided into two groups according to risk score, were performed on GO gene sets. The study included 1000 random sample permutations and enriched gene sets with a nominal *p* < 0.05and FDR < 0.25 were considered statistically significant. All other parameters were set based on their default values.

## Results

### Data source and processing

Initially, we obtained a total of 14143 lncRNA expression and 19659 gene expression profiles from 1053 breast cancer samples and 111 normal samples. In addition, the corresponding clinical data of 986 patients were downloaded from TCGA. The immune-related gene list downloaded from the ImmPort database contained 1534 immune related genes (Additional file 1: Table S1). We then obtained 937 immune-related lncRNAs through co-expression analysis of the immune gene list (*p* < 0.001). The top 10 positively and negatively correlated lncRNAs are shown sorted by correlation coefficient in Table 1.

**Table 1:** Top 10 positive/negative immune-related lncRNAs^1^

immuneGene	lncRNA	correlation coefficient	*p *value	Regulation
CD19	AC243960.1	0.931523591	0	Positive
CD79B	AC243960.1	0.921700762	0	Positive
TNFRSF13C	LINC00926	0.91156984	0	Positive
CD3D	AC004585.1	0.906199227	0	Positive
CD19	LINC00926	0.902280178	0	Positive
LCK	AC004585.1	0.901867543	0	Positive
PTPRC	AL365361.1	0.892830584	0	Positive
ZAP70	AC243960.1	0.892093262	0	Positive
CD3E	AC004585.1	0.889534261	0	Positive
CD48	LINC01857	0.888856061	0	Positive
UBXN1	OIP5-AS1	−0.4915959	3.67E−65	Negative
NFKBIB	OIP5-AS1	−0.476266029	1.00E−60	Negative
NFATC3	SPINT1-AS1	−0.468085147	1.89E−58	Negative
NCK2	AC008771.1	−0.467026674	3.69E−58	Negative
IGF2R	AC073896.4	−0.46300298	4.58E−57	Negative
UBR1	AP001505.1	−0.449374123	1.81E−53	Negative
CBL	SPINT1-AS1	−0.448571697	2.91E−53	Negative
PSMC3	AL122035.1	−0.448372597	3.28E−53	Negative
IFNAR2	AC008771.1	−0.446906674	7.78E−53	Negative
HSPA8	AC108673.3	−0.443217552	6.75E−52	Negative

1 represents sorted by correlation coefficient

### Identification of immune-related lncRNAs and construction of the prognostic model

**Fig. 1:**
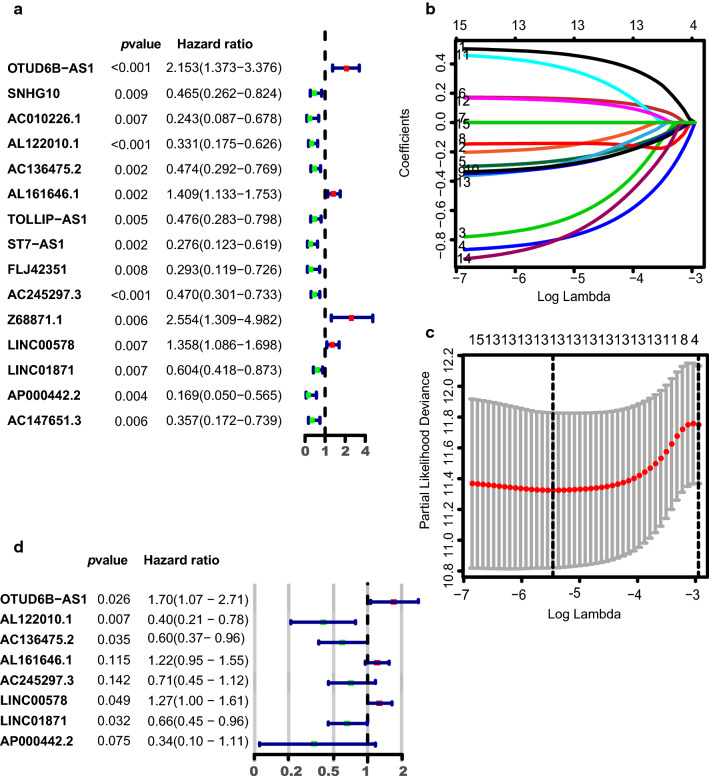
Identification and construction of the immune-related lncRNAs prognostic model by univariate Cox regression and Lasso regression analysis. **a **Forest plot of 15 candidate immune-related lncRNAs selected by univariate Cox regression analysis associated with breast cancer survival in the training set. **b** LASSO coefficient profiles of the 15 candidates in the training set. **c** A coefficient profile plot was generated against the log (lambda) sequence. Selection of the optimal parameter (lambda) in the LASSO model. **d** Forest plot of 8 candidate immune-related lncRNAs Selected by LASSO regression analysis associated with breast cancer survival and construction prognostic model

The data of breast cancer patients was allocated randomly to the training and validation cohort, 494 patient samples in the training cohort, 492 patient samples in the validation cohort. We carried out univariate Cox regression analysis on the expression profiles of the lncRNAs in the training set and obtained 15 candidate immune-related lncRNAs, significantly associated with survival, *p* < 0.01(Fig. 1a, Table 2). We performed Lasso regression on these lncRNAs. In order to avoid over fitting of the predicted signal, the prediction accuracy wasestimated by tenfold cross-validation (Fig. 1b, c**)**. A total of 8 immune-related lncRNAs were obtained, including OTUD6B-AS1(HR = 1.698; 95% CI 1.066–2.707, *p* = 0.026), AL122010.1(HR = 0.404; 95% CI 0.209–0.782, *p* = 0.007), AC136475.2(HR = 0.596; 95% CI 0.369–0.964, *p* = 0.035), AL161646.1(HR = 1.215; 95% CI 0.954–1.549, *p* = 0.115), AC245297.3(HR = 0.710;95% CI 0.450–1.121, *p* = 0.142), LINC00578(HR = 1.269; 95% CI 1.001–1.609, *p* = 0.049), LINC01871(HR = 0.657; 95% CI 0.448–0.964, *p* = 0.032), AP000442.2 (HR = 0.335; 95% CI 0.101–1.115, *p* = 0.075) (Fig. 1d, Table 3). So OTUD6B-AS1, AL161646.1 and LINC00578 were risk factors with HR > 1, while AL122010.1, AC136475.2, AC245297.3, LINC01871 and AP000442.2 were protective factors with an HR < 1. The expression of all the 8 immune-related lncRNAs in breast cancer was shown in the Additional file 2: Figure S1 and Additional file 3: Table S2, then we compared their expression between cancer samples and normal samples (Additional file 4: Figure S2). Except for AL161646.1 and LINC00578 (*p* < 0.0001), lncRNAs, including OTUD6B-AS1, AC245297.3, AC136475.2, AL122010.1, AL161646.1 and LINC01871, were high expressed between breast cancers and normal tissues. Some of the correlation between the lncRNAs and immune genes were shown in Additional file 5: Figure S3. The risk score for each sample was calculated based on the expression levels of these 8 lncRNAs. Risk score = (0.53* OTUD6B-AS1) + (−0.91* AL122010.1) + (−0.52* AC136475.2) + (0.20* AL161646.1) + (−0.34* AC245297.3) + (0.24* LINC00578) + (−0.42* LINC01871) + (−1.10* AP000442.2) (Table 3).

**Table 2:** Univariate Cox analysis for overall survival of 15 immune-related LncRNAs in training set

ID	HR	HR.95L	HR.95H	*p*value
OTUD6B-AS1	2.153122116	1.373184702	3.376046091	0.000832119
SNHG10	0.464651236	0.261933882	0.824256754	0.008771445
AC010226.1	0.243245961	0.087245203	0.678187397	0.00688694
AL122010.1	0.330788811	0.174928898	0.625518362	0.000665713
AC136475.2	0.473639558	0.291879867	0.768584807	0.002481537
AL161646.1	1.409383671	1.132997857	1.753191605	0.00206214
TOLLIP-AS1	0.475597922	0.283403961	0.798130634	0.004898673
ST7-AS1	0.276461931	0.123420958	0.619272451	0.001780562
FLJ42351	0.293301101	0.118560825	0.725581453	0.007952261
AC245297.3	0.46968286	0.300849229	0.733264265	0.000884015
Z68871.1	2.553691945	1.308915791	4.982247593	0.005970224
LINC00578	1.358183537	1.086209076	1.698257324	0.007246749
LINC01871	0.604154147	0.418198337	0.87279695	0.007256751
AP000442.2	0.168810321	0.050411344	0.565287931	0.003913707
AC147651.3	0.356585393	0.172081652	0.738911677	0.005538818

**Table 3:** Construction of 8 immune-related lncRNAs prognostic signature

ID	Correlation coefficient	HR	HR.95L	HR.95H	*p *value
OTUD6B-AS1	0.529692531	1.69841002	1.065676476	2.706822063	0.025916888
AL122010.1	−0.905807602	0.404215308	0.209034782	0.781640328	0.007098656
AC136475.2	−0.517003209	0.596304874	0.368979652	0.963683231	0.034771353
AL161646.1	0.195137911	1.215478602	0.953505844	1.549427559	0.115127418
AC245297.3	−0.342477733	0.710008929	0.449757063	1.120855504	0.141510647
LINC00578	0.238157741	1.268909336	1.000738329	1.608942972	0.049292045
LINC01871	−0.419944116	0.657083539	0.447993222	0.963761851	0.031647378
AP000442.2	−1.093407536	0.335072774	0.100718288	1.114730656	0.074608365

### The immune-related lncRNA model is a robust prognostic tool for breast cancer

**Fig. 2:**
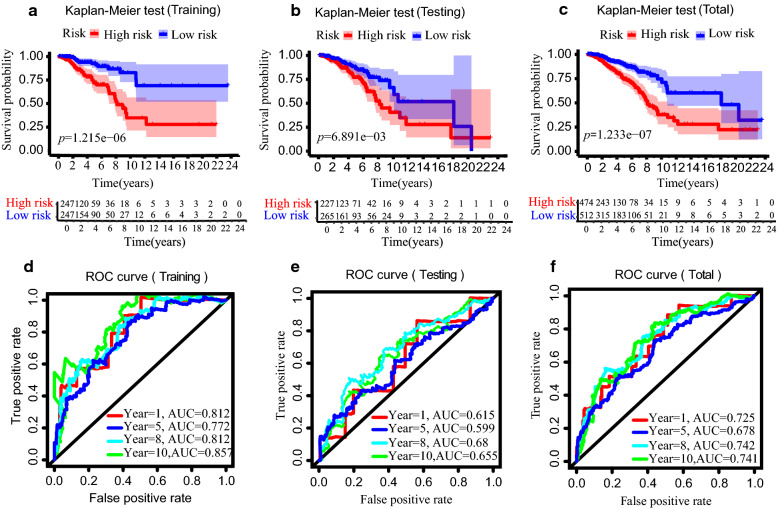
Verification of immune-related lncRNAs prognostic signature’s prediction ability. Kaplan–Meier survival analysis for the overall survival curves of breast cancers in the training (**a**), testing (**b**) and total set (**c**) with a low or high risk of death, according to the model based classifier risk score level. Time‐dependent receiver operating characteristic (ROC) analysis of the sensitivity and specificity of the survival for the immune-related lncRNAs risk score in the training (**d**), testing (**e**) and total set (**f**)

Breast cancer patients were divided into low- and high-risk groups according to the median risk score in the training set. Figure 2a presents the result of the Kaplan–Meier test. The *p* value of the log-rank test was 1.215e − 06, indicating that patients in the low-risk group had a 10 year longer median OS compared with the high-risk group. To assess the accuracy of the prognostic model, further examinations in both the testing set and the whole set were performed with the same algorithm cutoff. Both sets yielded similar results. The low-risk group exhibited remarkably better overall survival (OS) than the high-risk group, which indicated that the prognostic signature was effective (*p* = 0.0069 in the validation set; *p* = 1.233e − 07 in the total set) (Fig. 2b, c).

The ROC curve analysis of the model in the training set demonstrated its promising predictive value for breast cancer survival (1-year AUC = 0.812, 5-year AUC = 0.772, 8-year AUC = 0.81, 10-year AUC = 0.857, Fig. 2d). We then validated the model in the testing set, and the 1-year AUC was 0.615, the 5-year AUC was 0.599, the 8-year AUC was 0.68, and the 10-year AUC was 0.655 (Fig. 2e). As for the total cohort, the 1-year AUC was 0.725, the 5-year AUC was 0.678, the 8-year AUC was 0.742,and the 10-year AUC was 0.741 (Fig. 2f).

Principal component analysis (PCA) of the training, testing, and total breast cancer cohort demonstrated a different distribution pattern between the high- and low-risk groups, based on the expression of the 8 immune-related lncRNAs. This was indicative of the difference between the immune phenotypes of the groups (Additional file 6: Figure S4).

### Assessment of the correlation between candidate lncRNAs and clinicopathological characteristics

**Fig. 3:**
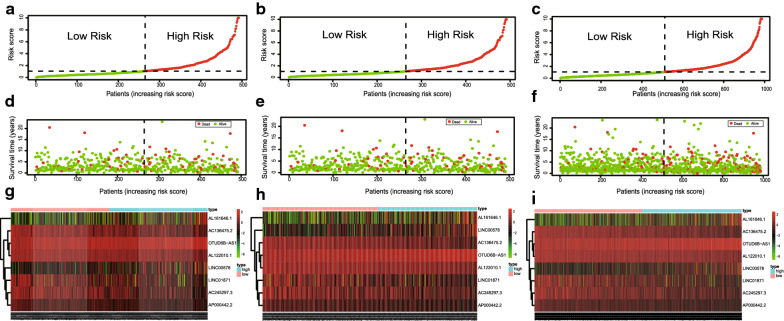
Immune-related lncRNA signature risk score analysis. The signature risk score distribution in the training (**a**), testing (**b**) and total set (**c**). The scatter plot of the sample survival overview in the training (**d**), testing (**e**) and total set (**f**), the green and red dots respectively represent survival and death. Heatmap showed the expression profiles distribution of the signature in the low-risk groups and high-risk groups in the training (**g**), testing (**h**) and total set (**i**), the pink bar represented the low-risk group, and the blue bar represents the high-risk group

We generated risk curves and scatter plots to display the risk score and survival status of each breast cancer patient, not only in the training set, but also in the testing and in the total set. The risk coefficient and mortality in the low-risk group were lower than those in the high-risk group (Fig. 3a–f).

Tumors with high prognostic scores expressed high-risk immune-related lncRNAs, whereas tumors with low prognostic scores expressed protective immune-related lncRNAs. The heatmap revealed that OTUD6B-AS1, AL161646.1, and LINC00578 were highly expressed in the low-risk group, while AC136475.2, AL122010.1, LINC01871, and AP000442.2 were highly expressed in the high-risk group (Fig. 3g–i).

**Fig. 4:**
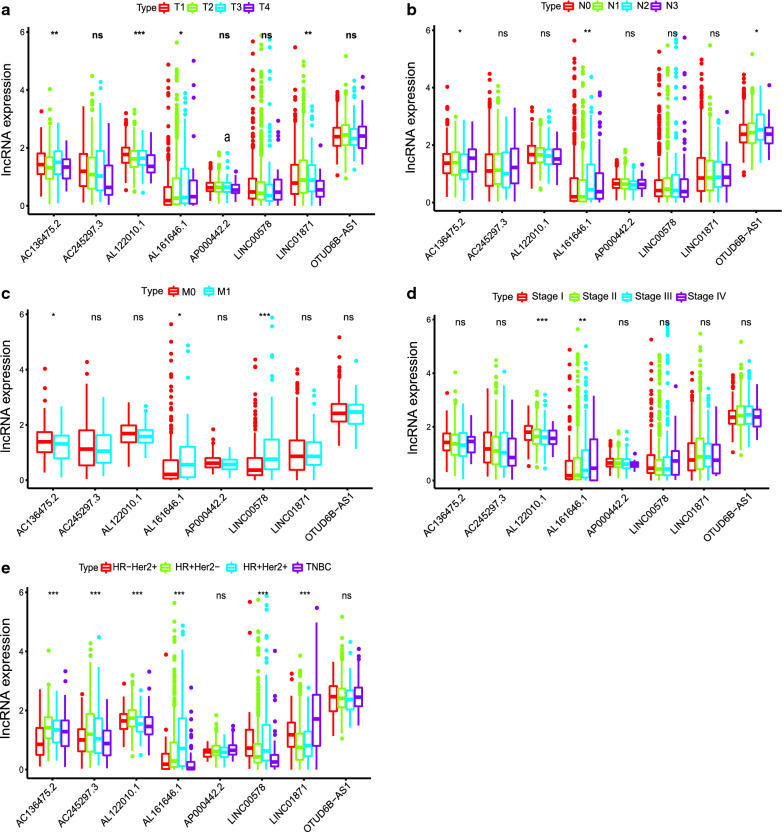
Correlations between risk score of the 8 immune-related lncRNAs-based model with clinicopathological characteristics. The box-plot showed that there were statistical difference expressions of the candidate immune-related lncRNAs in T (**a**) N (**b**), molecular typing (**c**) in the whole cohort

Further, in the overall sample, we analyzed the relationship between the expression of the 8 candidate lncRNAs and different clinicopathological factors (such as T, N, M, stage of 7th AJCC, molecular typing, etc.). Our results confirmed that differential expression of AC136475.2 (*p* < 0.01), AL122010.1 (*p* < 0.001), AL161646.1 (*p* < 0.05), LINC01871 (*p* < 0.01) could be observed among different T grades (Fig. 4a). The differences in expression of AC136475.2 (*p* < 0.05), AL161646.1 (*p* < 0.01), and OTUD6B − AS1 (*p* < 0.05) were statistically significant between different N groups (Fig. 4b). High expression of AL161646.1 (p < 0.05) and LINC00578 (*p* < 0.001) was observed in M1 group, while low expression of AC136475.2 was observed in M1 group (p < 0.05) (Fig. 4c). The differences in expression of AL122010.1 (*p* < 0.001) and AL161646.1 (p < 0.01) were statistically significant between different stage groups (Fig. 4d). Except for AP000442.2 and OTUD6B − AS1, lncRNAs, including AC136475.2, AC245297.3 (*p* < 0.001), AL122010.1 (*p* < 0.001), AL161646.1 (*p* < 0.001), LINC00578 (*p* < 0.001), and LINC01871 (*p* < 0.001), were differently expressed between different breast cancer molecular types (Fig. 4e).

### Evaluation of the immune-related lncRNA signature as an independent prognostic factor in patients with breast cancer

**Fig. 5:**
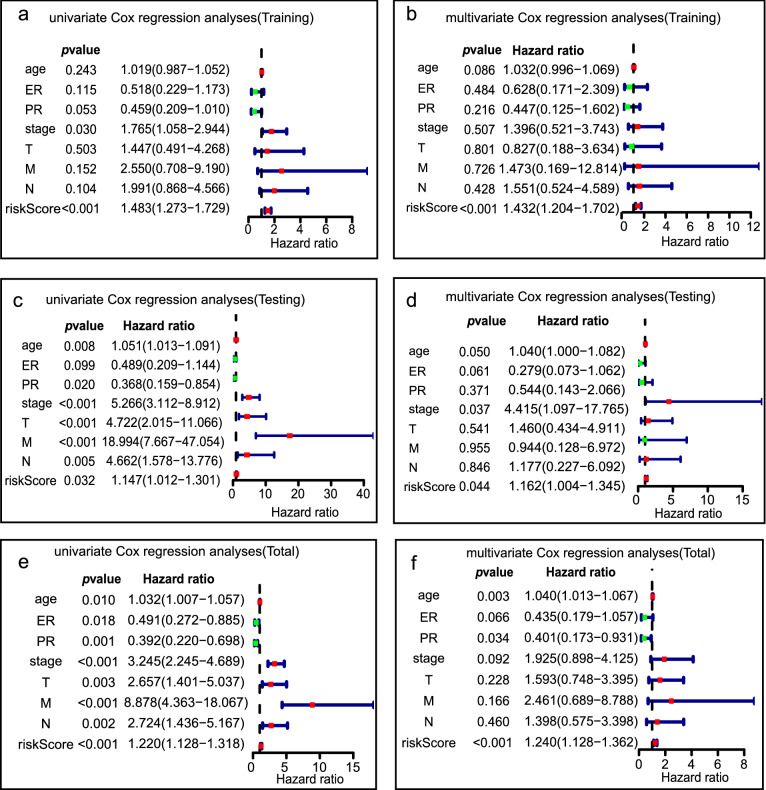
The Cox regression analysis for evaluating the independent prognostic value of the risk score. Univariate (**a**) and multivariate Cox regression analyses (**b**) of the model in the training set. Univariate (**c**) and multivariate Cox regression analyses (**d**) of the model in the testing set. Univariate (**e**) and multivariate Cox regression analyses (**f**) of the model in the total set

We carried out univariate and multivariate Cox regression analyses to verify that the model could serve as an independent prognostic factor for breast cancer, also accounting for certain clinicopathological variables (such as age, ER status, PR status, AJCC 7th T stage, etc.) (Fig. 5). The univariate Cox analysis revealed that the high-risk group was significantly correlated with shorter survival in the training set (HR = 1.483; 95% CI 1.273–1.729, *p* < 0.001), validation set (HR = 1.147; 95% CI 1.012–1.301, *p* = 0.032), and whole set (HR = 1.220; 95% CI 1.128–1.318, *p* < 0.001). Multivariate Cox regression analyses of the above mentioned factors indicated that the immune-related lncRNA model was a reliable and independent prognostic factor for OS in the training set (HR = 1.432; 95% CI 1.204–1.702, *p* < 0.001), validation set (HR = 1.162; 95% CI 1.004–1.345, *p* = 0.044), and whole set (HR = 1.240; 95% CI 1.128–1.362, *p* < 0.001). In the whole set, multivariate analysis revealed that age (HR = 1.040; 95% CI 1.013–1.067, *p* = 0.003) and PR status (HR = 0.401; 95% CI 0.173–0.931, *p* = 0.034) were independent prognostic factors for OS.

### Gene set enrichment analysis for functional annotation of the immune-related risk signature

**Fig. 6:**
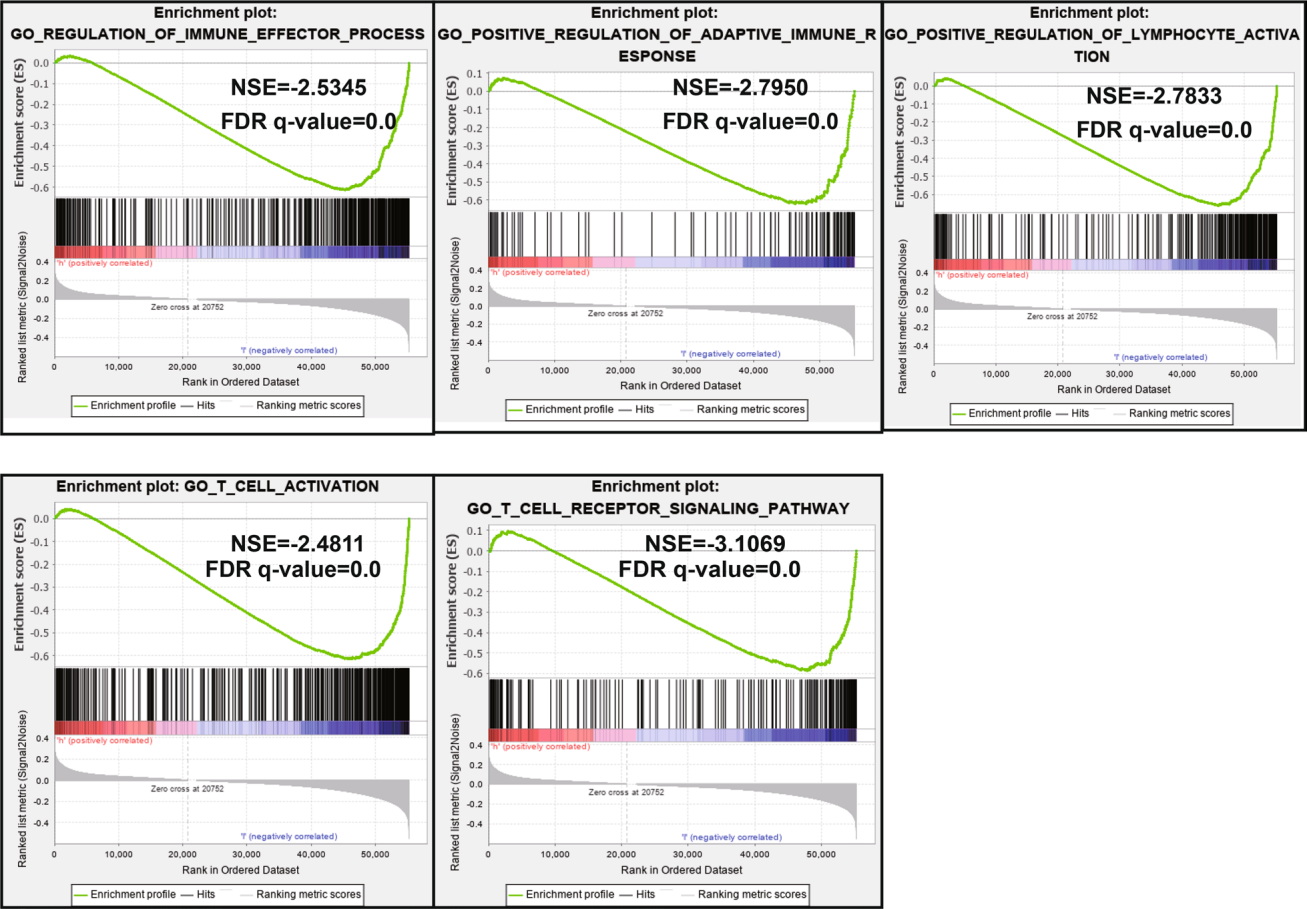
GSEA analysis of the differentially expressed genes between high and low risk groups.

GSEA of the risk signature was performed using the GSEA software. The results revealed that immune-related responses were further enriched in low-risk groups compared to high-risk groups. We demonstrated 5 immune-related gene ontology terms in the GSEA results with FDR < 0.25 (Fig. 6), including the positive regulation of immune effector processes, positive regulation of the adaptive immune response, positive regulation of lymphocyte activation, regulation of T cell activation, and the T cell receptor signaling pathway.

## Discussion

With the in-depth researches on lncRNAs and the immune system [21–24], scholars have realized that immune-related lncRNAs may prove to be useful not only as potential prognostic biomarkers but could also provide novel therapeutic options. Nevertheless, the lack of validation cohorts is a limitation for the proper evaluation of the prognostic merit of potential biomarkers. In the current study, we identified a novel immune-related lncRNA molecular signature using Cox and Lasso regression analyses. The signature was then validated in a testing group and a total group, indicative of its robustness and reliability. The signature demonstrated good predictive performance and could effectively classify breast cancer patients into a high-risk and a low-risk group within the training and validation sets. The low-risk group had a significantly longer overall survival compared with the high-risk group. Further, the signature proved to be an independent prognostic factor based on multivariate Cox analysis, which revealed the signature’s reproducibility and reliability for breast cancer prognosis.

Among the 8 candidate lncRNAs, AC136475.2, AL161646.1, LINC01871, and AP000442.2 had not been previously reported. OTUD6B-AS1 and LINC00578 were discovered as prognostic signatures in breast cancer for the first time. Although AL122010.1 and AC245297.3 had been previously reported as prognostic signatures in breast cancer [25], our research characterized their functional involvement as lncRNAs associated with tumor immunity. OTUD6B-AS1 is transcribed from the opposite strand of the OTUD6B gene, which is located on chromosome 8 in head-to-head orientation to OTUD6BAS1 [26]. It was reported that high OTUD6B-AS1 expression indicates poor prognosis in ovarian cancer [27]. However, Gang Wang et al*. *[28] found that high OTUD6B-AS1 expression was associated with improved survival and inhibited clear cell renal cell carcinoma proliferation via the Wnt/β-catenin signaling pathway. Zhuolu Wang et al. found that OTUD6B-AS1 inhibits viability, migration, and invasion of Thyroid Carcinoma by Targeting miR-183-5p and miR-21 [29]. Further more, it was reported that the expression of miR-21 in cells of the tumor immune infiltrate, and in particular in macrophages, was responsible for promoting tumor growth [30]. Activation-induced up regulation of miR-21 biases the transcriptome of differentiating T cells away from memory T cells and toward inflammatory effector T cells [31]. In the current study, we found that the overall survival was lower under high OTUD6B-AS1 expression. Further, expression was higher in the N1 and N2 groups when compared to the N0 group. We speculated that OTUD6B-AS1 affects macrophages and differentiating T cells through miR-21, then affects immune status, and finally affects the occurrence and development of tumor. Therefore, further research and mechanistic insight are required. LINC00578 was found as a potential biomarker in lung adenocarcinoma [32, 33], major depressive disorder [34], and pancreatic cancer [35]. Although the AC243960.1 and OIP5-AS1 lncRNAs were the most positively and negatively correlated, respectively, with immune genes, they did not appear in the signature. It was indicated that high AC243960.1 expression indicates better prognosis in breast cancer, however, no statistically significant interactions between OIP5-AS1 expression and OS was found (Additional file 7: Figure S5). This may be due to the deletion of some relevant data during regression analysis.

As breast cancer is a highly heterogeneous disease, scientists divide breast cancers into different clinically relevant molecular subtypes based on the expression levels of the estrogen receptor (ER), progesterone receptor (PR), and HER2 [36–38]. Distinct prevalence, prognosis, and systemic therapies are utilized in the management of these different breast cancer subtypes [39–41]. HER2-overexpressing (ER and PR− , HER2 +) and triple-negative (ER and PR− , HER2−) subtypes are known to be more aggressive and have poorer outcomes [38, 42]. Our results indicated that the expression of LINC01871 was high on both subtypes. Meanwhile, and LINC01871 shows a strong positive correlation with immune genes such as GZMB, CTLA4, PDCD1 etc. (Additional file 5: Figure S3). GZMB, the most potent cytotoxic molecules, act mainly as antitumoral and anti-infectious factors. However, when expressed by immune regulatory cells it may contribute to immune evasion of specific cancer types [43]. Cytotoxic T lymphocyte-associated antigen 4 (CTLA4) and programmed death-1 (PD-1; encoded by the PDCD1 gene) represent crucial immune checkpoints, the blockade of which can potentiate anti-tumour immunity [44, 45]. Therefore, it is suggested that LINC01871 may play an important role, particularly related to the above immune processes and immune genes, in the development of breast cancer in the two phenotypes, which requires in-depth investigation in the future.

Finally, GSEA further confirmed the robust connection of the signature with the immune response. Samples from patients with low-risk scores were associated with positive regulation of adaptive immune response. Further, patients with a high-risk score exhibited greater adaptive immune resistance. Adaptive immune resistance is a process during which cancers change their phenotype in response to a cytotoxic or pro-inflammatory immune response, thereby evading it [46]. Inhibition of adaptive immune resistance is the mechanistic foundation of responses to PD-1 or PD-L1 inhibition [47, 48], which has made a significant contribution to the treatment of breast cancer [49, 50]. Our prognostic signature may provide directions for predicting the efficacy or studying the mechanism of PD-1 or PD-L1 inhibition immunotherapy. Lymphocytes, including T cells, B cells, and natural killer cells, are the main force underlying immune defense mechanisms. For them to perform their immune function, lymphocytes must be activated either through the recognition and binding of antigens or through stimulation by cytokines [51–53]. In this study, samples from patients with low-risk scores were associated with the positive regulation of lymphocyte activation, which may indicate that low-risk patients have a more active immune status and better immune defense than do high-risk patients. T cells have been considered as having a significant role in immune surveillance and tumor eradication. On the basis of this paradigm, over the past quarter century, T cell-based cancer therapies have achieved success in patients [54, 55].

The advantage of the current study was that our signature is based on population databases and high-throughput sequencing data. Further, both exploration and validation were used in order to evaluate the risk score method. Tumor immunology has become the most rapidly advancing area of cancer research, and immunotherapy has provided promising treatment in recent years. The current study utilized a new immune-related prognostic approach for breast cancer. However, there were some limitations in this study. First, as the analyzed data was obtained from online databases, the study was of retrospective nature. Second, there is no in vitro or in vivo experimental data confirming our findings. In addition, we did not explore the potential mechanisms of investigated lncRNAs. Thus, more functional studies on the 8 lncRNAs, alone and in combination, should be carried out to further test the predictive accuracy of the signature and discover potential immune-related mechanisms. Of note, we are currently working on the clinical validation and mechanistic elucidation of these results. In various studies, gene expression differences between cancer and normal tissues are compared for the screening of prognostic genes. This may leave out certain genes with little expression differences between cancer and normal tissues. Such genes with no obvious expression difference may have a great influence on the biological behavior of tumors, chemotherapy, immunotherapy, and other factors affecting the survival of patients. Despite these limitations, to the best of our knowledge, this study is the first to report the external validation of an established immune-related lncRNA signature for breast cancer. The eight immune-related lncRNAs had never been studied in breast cancer.

## Conclusions

In conclusion, we have identified an 8 immune-related lncRNA signature as a potential prognostic tool for breast cancer patients. It is strongly connected to the risk value, tumor status, and OS. The signature provides a novel insight into immune-related lncRNAs in breast cancer and identifies potential biomarkers for prognosis and immunotherapy.

## Supplementary information

**Additional file 1: Table S1.** The immune-related gene list downloaded from the ImmPort database.

**Additional file 2: Figure S1.** The 8 immune-related lncRNAs expression in breast cancers.

**Additional file 3: Table S2.** The 8 immune-related lncRNAs expression in breast cancers.

**Additional file 4: Figure S2.** The difference expression of the 8 immune-related lncRNAs between cancer samples and normal samples.

**Additional file 5: Figure S3.** The correlation between the lncRNAs and immune genes.

**Additional file 6: Figure S4.** Principal component analysis of the training, testing, and total set with the 8 immune-related lncRNAs signature.

**Additional file 7: Figure S5.** Kaplan-Meier survival analysis for the overall survival curves of breast cancers in the total set.

## Data Availability

The datasets analysed during the current study are available in the TCGA repository (https://cancergenome.nih.gov/).

## Abbreviations

lncRNA: Long non-coding RNA
TCGA: The Cancer Genome Atlas
OS: Overall survival
LASSO: Least absolute shrinkage and selectionoperator
ROC: Receiver operating characteristic
AUC: Area under the respective ROC
GSEA: Gene set enrichment analysis
PCA: Principal component analysis
HR: Hazard ratio
ER: Estrogen receptor
PR: Progesterone receptor
FDR: False discovery rate
