# Supplementary figures and images for: Immune-related lncRNAs as predictors of survival in breast cancer: a prognostic signature
Source: J Transl Med. 2020 Nov 23;18:442. doi: 10.1186/s12967-020-02522-6 (PMC7681988; doi:10.1186/s12967-020-02522-6)

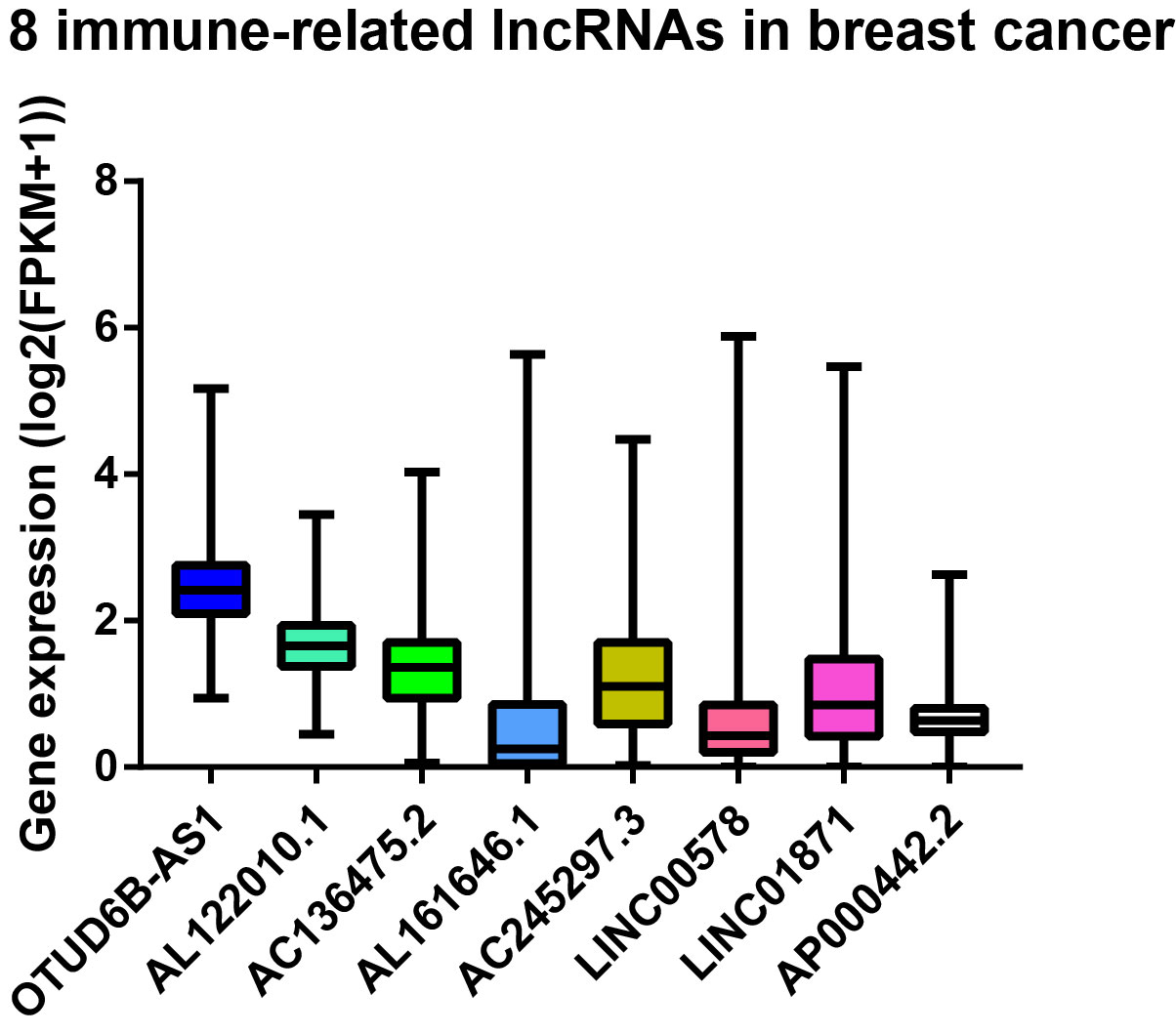

Supplement: Supplementary file 2 — Additional file 2: Figure S1. The 8 immune-related lncRNAs expression in breast cancers. [file 12967_2020_2522_MOESM2_ESM.jpg]

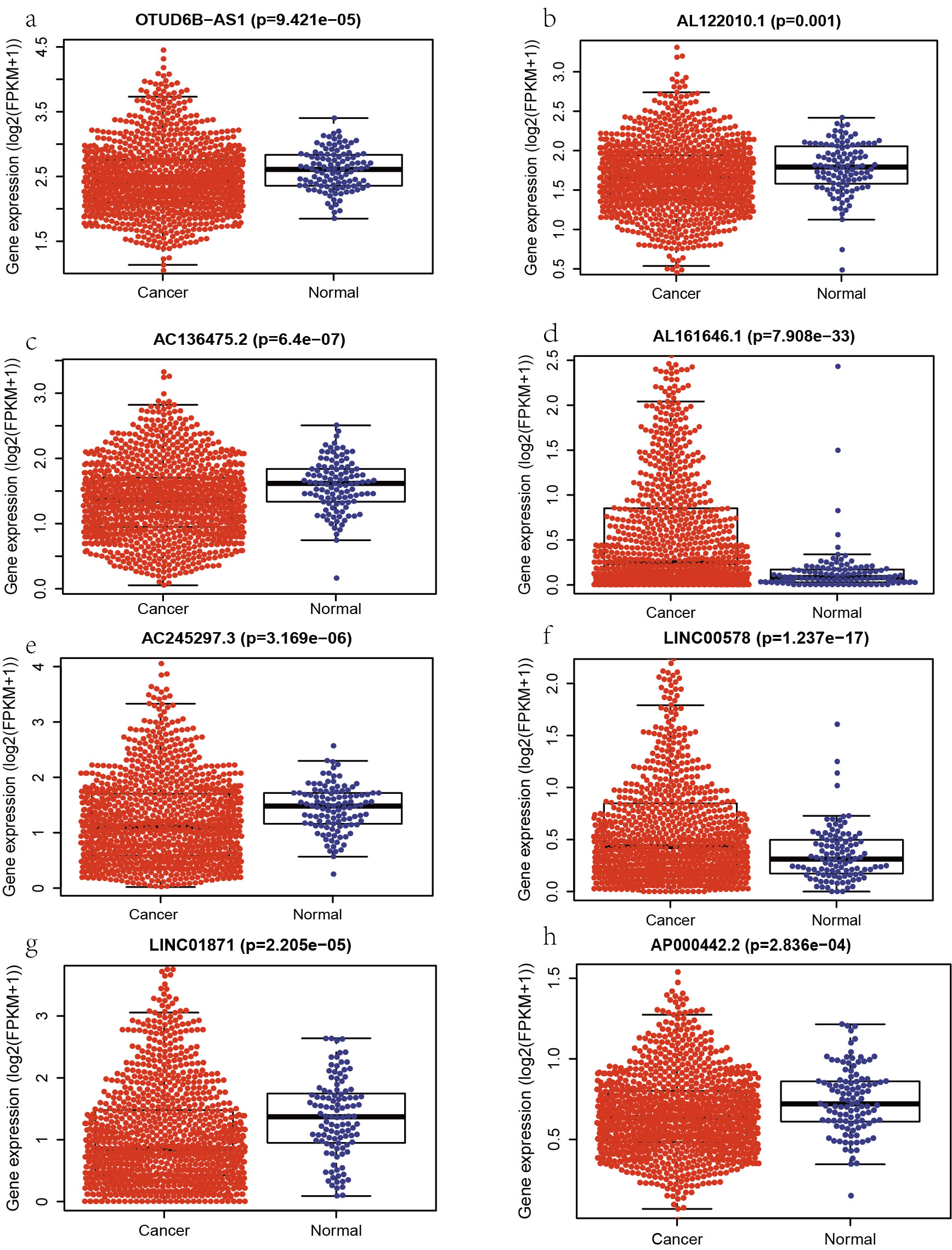

Supplement: Supplementary file 4 — Additional file 4: Figure S2. The difference expression of the 8 immune-related lncRNAs between cancer samples and normal samples. [file 12967_2020_2522_MOESM4_ESM.jpg]

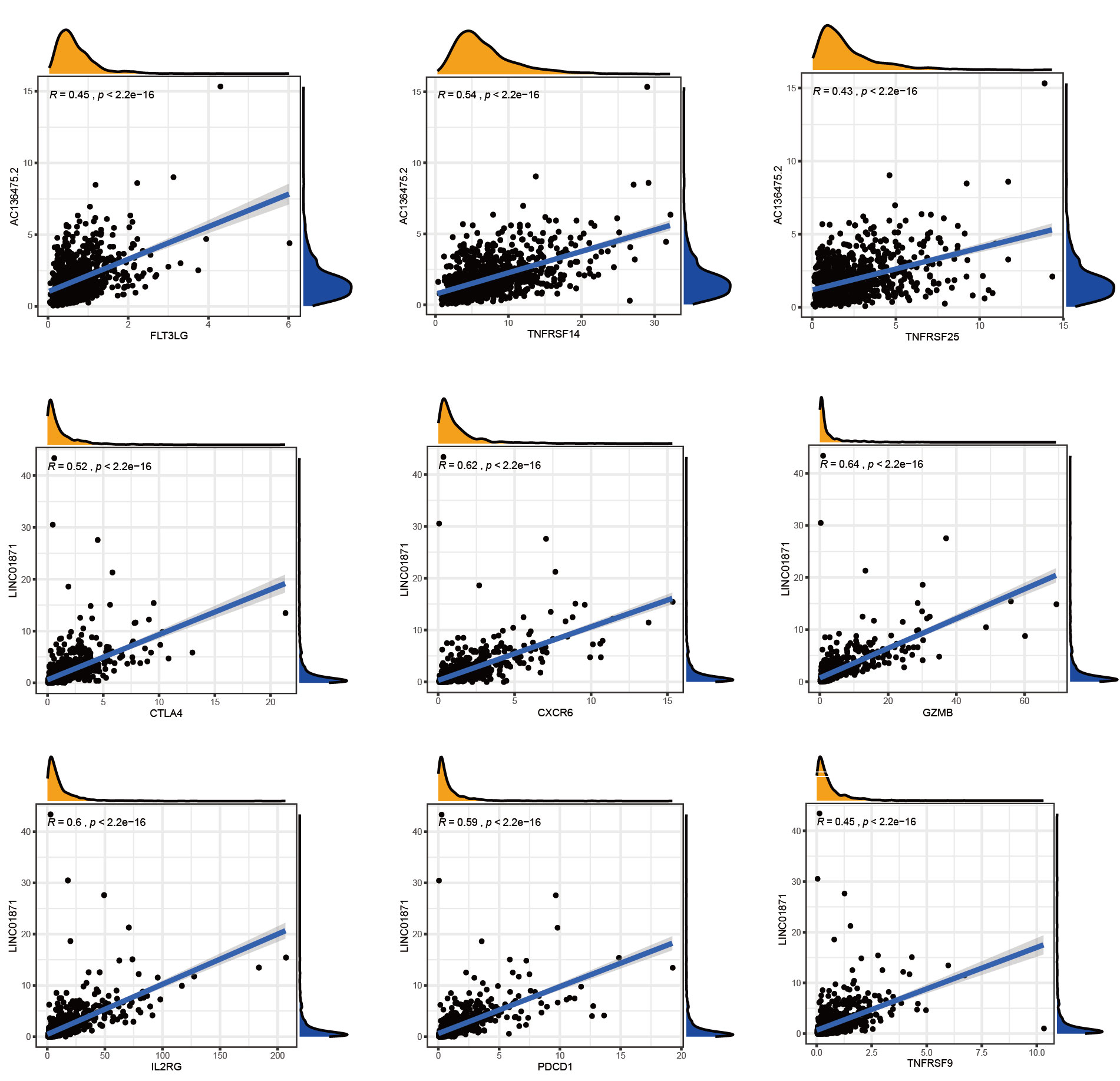

Supplement: Supplementary file 5 — Additional file 5: Figure S3. The correlation between the lncRNAs and immune genes. [file 12967_2020_2522_MOESM5_ESM.jpg]

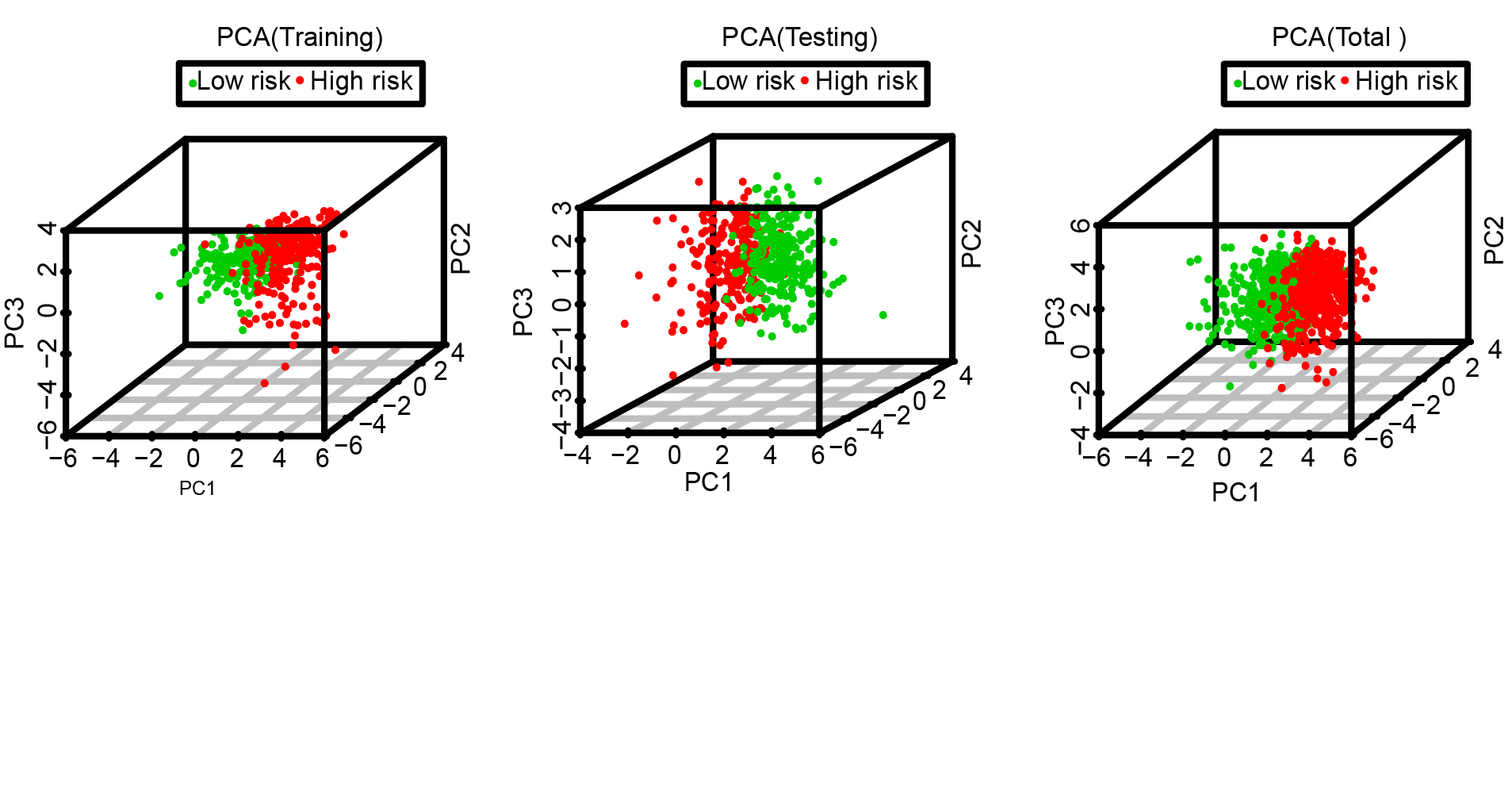

Supplement: Supplementary file 6 — Additional file 6: Figure S4. Principal component analysis of the training, testing, and total set with the 8 immune-related lncRNAs signature. [file 12967_2020_2522_MOESM6_ESM.jpg]

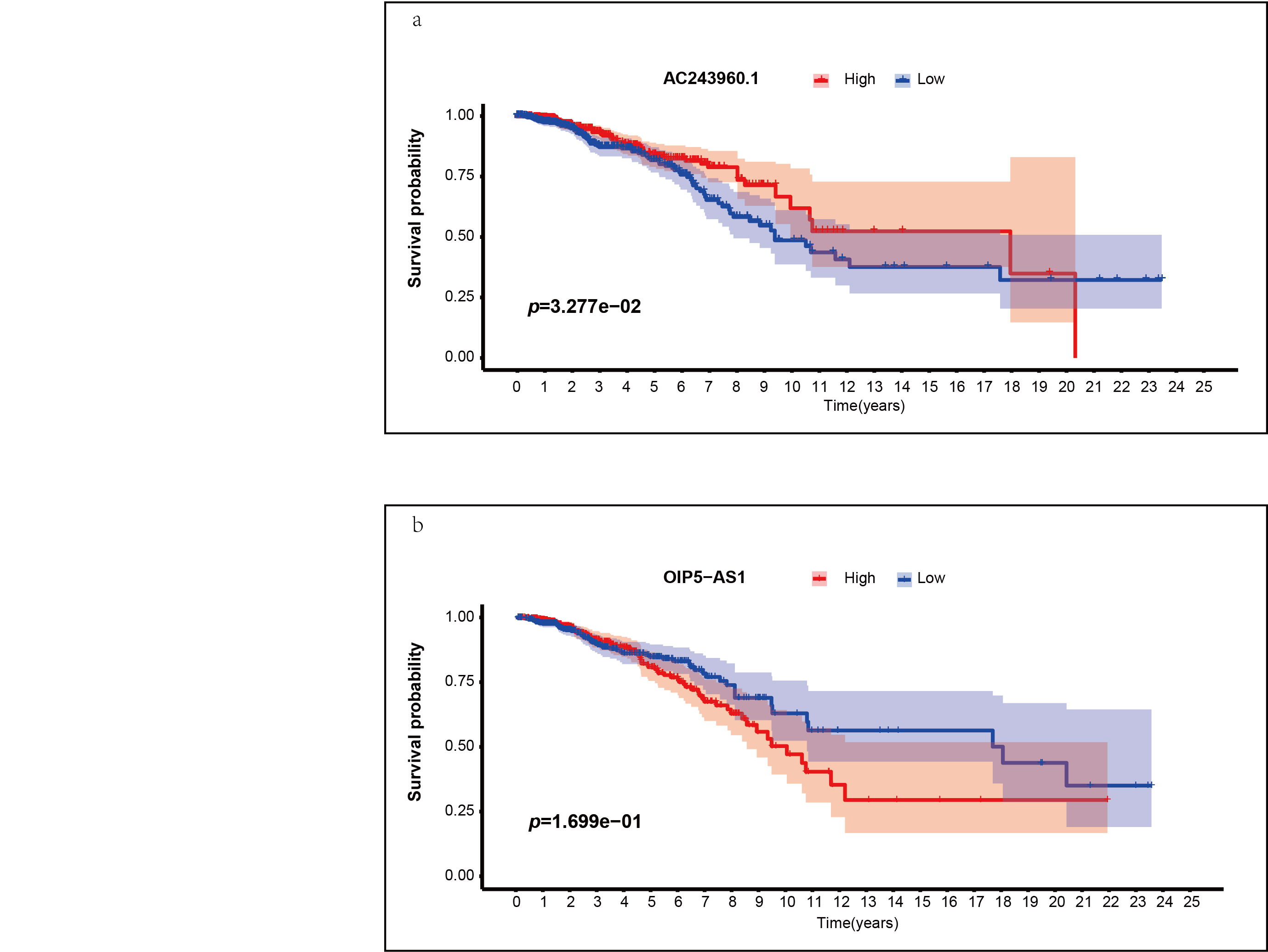

Supplement: Supplementary file 7 — Additional file 7: Figure S5. Kaplan-Meier survival analysis for the overall survival curves of breast cancers in the total set. [file 12967_2020_2522_MOESM7_ESM.jpg]
